# Exogenous Spermidine Alleviates Low Temperature Injury in Mung Bean (*Vigna radiata* L.) Seedlings by Modulating Ascorbate-Glutathione and Glyoxalase Pathway

**DOI:** 10.3390/ijms161226220

**Published:** 2015-12-17

**Authors:** Kamrun Nahar, Mirza Hasanuzzaman, Md. Mahabub Alam, Masayuki Fujita

**Affiliations:** 1 Laboratory of Plant Stress Responses, Department of Applied Biological Science, Faculty of Agriculture, Kagawa University, Miki-cho, Kita-gun, Kagawa 761-0795, Japan; knahar84@yahoo.com (K.N.); shamim1983@yahoo.com (M.M.A.); 2 Department of Agricultural Botany, Faculty of Agriculture, Sher-e-Bangla Agricultural University, Sher-e-Bangla Nagar, Dhaka-1207, Bangladesh; 3 Department of Agronomy, Faculty of Agriculture, Sher-e-Bangla Agricultural University, Dhaka-1207, Bangladesh

**Keywords:** abiotic stress, AsA-GSH cycle, glyoxalase system, oxidative stress, polyamine

## Abstract

The role of exogenous spermidine (Spd) in alleviating low temperature (LT) stress in mung bean (*Vigna radiata* L. cv. BARI Mung-3) seedlings has been investigated. Low temperature stress modulated the non-enzymatic and enzymatic components of ascorbate-glutathione (AsA-GSH) cycle, increased H_2_O_2_ content and lipid peroxidation, which indicate oxidative damage of seedlings. Low temperature reduced the leaf relative water content (RWC) and destroyed leaf chlorophyll, which inhibited seedlings growth. Exogenous pretreatment of Spd in LT-affected seedlings significantly increased the contents of non-enzymatic antioxidants of AsA-GSH cycle, which include AsA and GSH. Exogenous Spd decreased dehydroascorbate (DHA), increased AsA/DHA ratio, decreased glutathione disulfide (GSSG) and increased GSH/GSSG ratio under LT stress. Activities of AsA-GSH cycle enzymes such as ascorbate peroxidase (APX), monodehydroascorbate reductase (MDHAR), dehydroascorbate reductase (DHAR) and glutathione reductase (GR) increased after Spd pretreatment in LT affected seedlings. Thus, the oxidative stress was reduced. Protective effects of Spd are also reflected from reduction of methylglyoxal (MG) toxicity by improving glyoxalase cycle components, and by maintaining osmoregulation, water status and improved seedlings growth. The present study reveals the vital roles of AsA-GSH and glyoxalase cycle in alleviating LT injury.

## 1. Introduction

Low temperature (LT) or chilling temperature often adversely affects plant growth and productivity. Every year, plants covering a vast area of the world suffer from LT stress, which leads to substantial crop losses and thus LT stress is considered as one of the major abiotic stresses [1]. Low temperature stress causes physiological and metabolic disorder leading to reduced growth and vigor. Obstacles in plant–water relationships, reduced stomatal conductance, photosynthetic efficiency, changes in protein structure and enzyme activities are some of the most common and primary LT injury symptoms within plants [2]. Inhibition of photochemistry efficiency under LT stress increases generation of reactive oxygen species (ROS), which may include singlet oxygen (^1^O_2_), superoxide anion (O_2_^•−^), hydrogen peroxide (H_2_O_2_), and hydroxyl radical (OH^•^) [3]. ROS act as secondary messengers in signal transduction and play vital roles in plant growth and stress responses [4]. However, excess levels of ROS are responsible for cell membrane lipid peroxidation, protein denaturation, carbohydrate oxidation, photosynthetic pigment breakdown, impaired enzyme activity, and damage to nucleic acids. An excess amount of ROS is a threat for cell survival and often causes programmed cell death [5].

Maintaining a delicate balance between ROS generation and scavenging is important for plants, and is mainly accomplished by the antioxidant defense system. The antioxidant defense system is composed of the ascorbate-glutathione (AsA-GSH) cycle, consisting of two major non-enzymatic antioxidants, AsA and GSH, and four enzymes, ascorbate peroxidase (APX), monodehydroascorbate reductase (MDHAR), dehydroascorbate reductase (DHAR) and glutathione reductase (GR); these enzymes help AsA and GSH scavenge major ROS and regenerate in their active, reduced form through spontaneous biochemical reactions. Nonetheless, other major antioxidant enzymes linked to the AsA-GSH cycle are superoxide dismutase (SOD), catalase (CAT), and glutathione peroxidase (GPX), which also play crucial roles in the antioxidant system [3]. The toxic compound methyglyoxal (MG) production is an unavoidable consequence of the glycolysis pathway that is amplified under stress conditions. The common cytotoxic effects of MG include clogging up membrane structure and function, increasing ion leakage, deterioration of ultrastructural molecules and organelles, DNA damage or even cell death [6]. With the help of the glyoxalase pathway, plants reduce MG toxicity. The glyoxalase pathway consists of glyoxalase I (Gly I) and glyoxalase II (Gly II) enzymes. These two enzymes detoxify MG producing a less toxic compound using GSH as a cofactor [6,7].

Polyamines (PAs) are aliphatic polycation compounds ubiquitously found in living organisms; putrescine (Put), spermidine (Spd) and spermine (Spm) are the most common PAs. PAs are often termed as a new class of plant growth regulators due to their fundamental roles in physiological processes, including protein synthesis, cell-cycle regulation, ion-channel regulation, free-radical scavenging, signal transduction, gene expression, and many other not yet revealed metabolic processes where PAs are likely to have vital roles [8]. Thus, functions of PAs are crucial in plant growth, development, and adaptation to environmental stresses. PAs have a range of protective function in LT affected plants. The polycationic PAs can combine with cellular membrane and help to avoid intracellular ice formation. PAs enhance cellular antioxidant capacity, and decrease the peroxidation of unsaturated fatty acids during LT stress. PAs improve membrane stabilization and increase the fluidity [9]. Root application of PAs was proved to positively affect PSI and PSII (photosystem I and photosystem II, respectively) activities, and antioxidant systems, which conferred stress tolerance [10,11]. Plant acclimation, adaptation and tolerance to LT are often related to the production and changes of many potential phytoprotectant molecules including PAs [9]. Accumulation of PAs under LT stress was reported in a number of plant species. Low temperature acclimation and adaptation were achieved in plants by modulating the endogenous level of PAs and their biosynthetic or responsive genes [9,12,13]. Despite some existing reports regarding the roles of PAs under LT stress, the mechanism is not clear due to the complex nature of PAs, as PAs have diverse roles in plant physiology and biochemistry, where PAs not only function alone but also interact or boost up functioning of others like plant nutrients, osmoprotectants, antioxidant molecules, hormones, and signaling molecules [14].

Mung bean is a broadly cultivated legume crop having wide adaptability to different environmental conditions. Mung bean plant is grown in temperate regions and can be grown in all seasons throughout the year in tropical countries, where this plant may face LT or chilling stress in the winter [15,16]. To our knowledge, the spermidine mediated coordinated role of AsA-GSH and glyoxalase cycle in mitigating LT-induced oxidative stress in plants has not been reported previously. The present study was carried out to find the LT response of mung bean plants and to reveal the roles of exogenous Spd to regulate antioxidant system and glyoxalase cycle in conferring LT stress.

## 2. Results

### 2.1. Dry Weight of Seedlings

**Figure 1:**
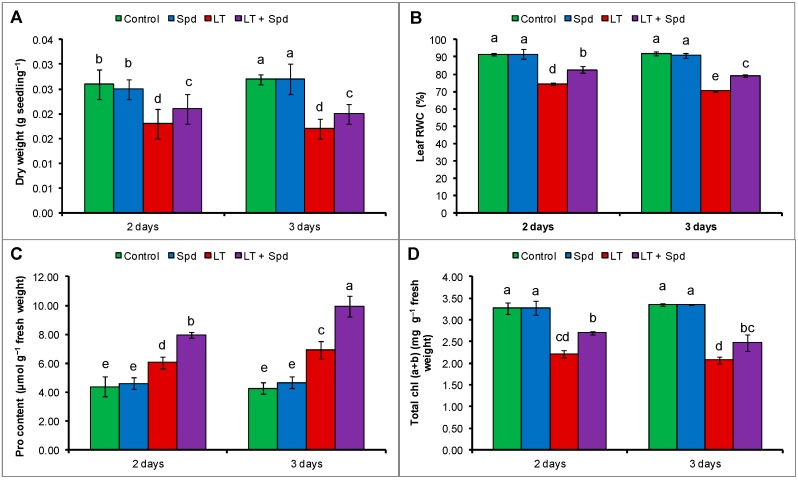
Dry weight per seedling (**A**); leaf relative water content (RWC) (**B**); proline (Pro) content (**C**); and total chlorophyll, chl (*a* + *b*) content (**D**) in mung bean seedlings. Spd and LT indicate spermidine (0.25 mM) and low temperature (6 °C), respectively. Mean (±SD) was calculated from three replicates for each treatment. Bars with different letters are significantly different at *p* ≤ 0.05 applying Duncan’s multiple range test (DMRT).

Low temperature reduced the growth of mung bean seedlings, which reduced the dry weight of seedlings. A reduction of dry weight per seedling was evident both after two days and three days of LT stress. Dry weight per seedling reduced by 31% after two days and it reduced by 37% after three days of LT stress, compared to the control. Exogenous Spd increased dry weight of LT affected seedlings, compared to the LT affected seedlings without Spd application (Figure 1A).

### 2.2. Leaf Relative Water Content (RWC) and Proline (Pro) Content

Leaf RWC content reduced by 19% and 23% after two and three days of LT stress, respectively, compared to the seedlings under control temperature. In contrast, proline (Pro) contents in mung bean seedlings increased slightly under LT stress. However, exogenous Spd supplementation in LT affected mung bean seedlings maintained osmotic regulation and water status of mung bean seedlings, which is indicated by significant increase in Pro content and leaf RWC (compared to the LT only affected plants) (Figure 1B,C).

### 2.3. Leaf Chlorophyll Contents

Total chlorophyll, chl (*a* + *b*) of mung bean plants was reduced by 32% after two days of LT exposure, and after three days it was reduced by 38%, compared to the control seedlings. Again, compared to the LT affected seedlings, the seedlings pretreated with Spd recovered the reduction total chl (*a* + *b*) by increasing its content under LT stress (Figure 1D).

### 2.4. Histochemical Detection of Hydrogen Peroxide and Superoxide

**Figure 2:**
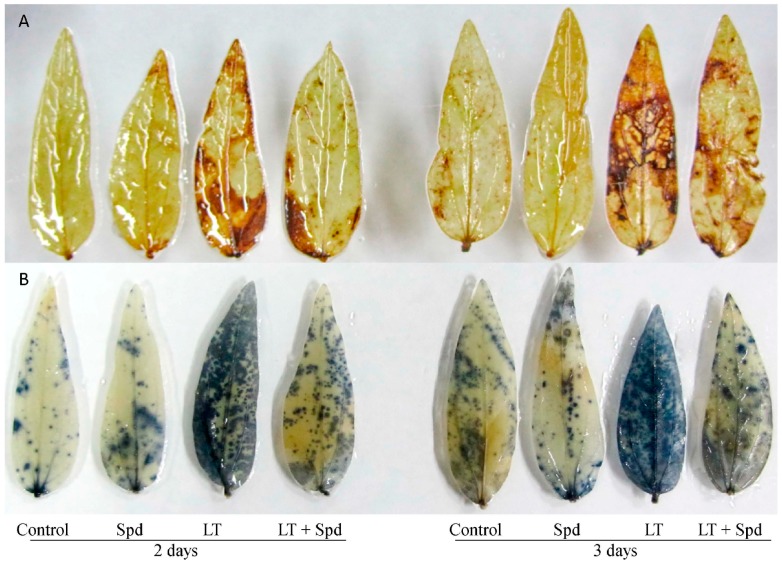
Histochemical localization of H_2_O_2_ (**A**); and O_2_^•−^ (**B**) in mung bean leaves. Spd and LT indicate spermidine (0.25 mM) and low temperature (6 °C), respectively.

Histochemical staining localized the spots of H_2_O_2_ and O_2_^•−^ at the subcellular level in the leaves of the mung bean plants that were affected by LT temperature stress. However, exogenous Spd pretreatment reduced the spots of H_2_O_2_ and O_2_^•−^ from the leaves of LT affected plants (Figure 2A,B).

### 2.5. Oxidative Stress Reduction by Spd

**Figure 3:**
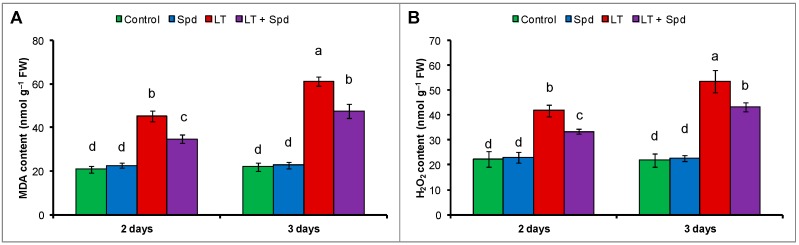
MDA (malondialdehyde, a product of lipid peroxidation) (**A**) and H_2_O_2_ (**B**) contents in mung bean seedlings. Spd and LT indicate spermidine (0.25 mM) and low temperature (6 °C), respectively. Mean (±SD) was calculated from three replicates for each treatment. Bars with different letters are significantly different at *p* ≤ 0.05 applying DMRT.

One of the primary indicators of LT stress was oxidative damage. Significant increase of H_2_O_2_ content and lipid peroxidation (malondealdehyde, MDA which is product of lipid peroxidation) was evident in LT affected mung bean seedlings. Preliminary experiment was conducted with different doses of Spd and 0.25 mM Spd resulted in the highest reduction of lipid peroxidation level (data has been presented as supplementary data in Figure S1). Lipid peroxidation increased by 117% and 179% after two days and three days of LT stress, respectively (Figure 3A) (compared to the control). Two and three days of LT stress increased the H_2_O_2_ contents by 88% and 145%, respectively, compared the control (Figure 3B). Exogenous Spd pretreatment reduced the oxidative stress by decreasing H_2_O_2_ content and lipid peroxidation (Figure 3A,B).

### 2.6. Ascorbate-Glutathione Cycle Components

#### 2.6.1. Non-Enzymatic Antioxidants

**Figure 4:**
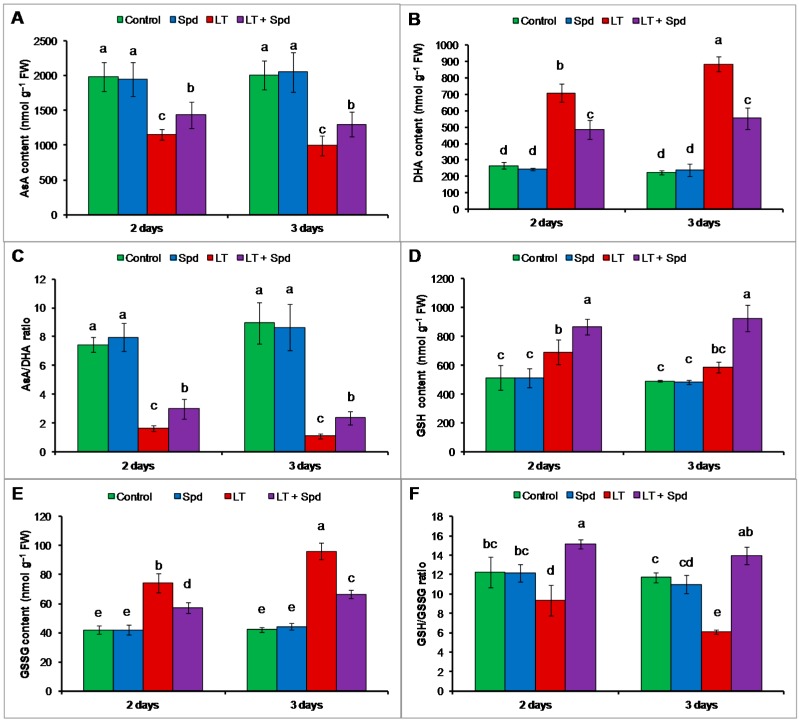
Ascorbate (AsA) (**A**) and dehydroascorbate (DHA) (**B**) contents; AsA/DHA ratio (**C**); glutathione (GSH) (**D**) and glutathione disulfide (GSSG) (**E**) contents; and GSH/GSSG ratio (**F**) in mung bean seedlings. Spd and LT indicate spermidine (0.25 mM) and low temperature (6 °C), respectively. Mean (±SD) was calculated from three replicates for each treatment. Bars with different letters are significantly different at *p* ≤ 0.05 applying DMRT.

The contents of AsA reduced and contents of DHA increased highly under LT stress, which reduced the AsA/DHA ratio (compared to the control) (Figure 4A–C). GSH and GSSG contents increased under LT stress but the GSH/GSSG ratio decreased under that stress condition (compared to the control) (Figure 4D–F). Exogenous Spd pretreatment increased AsA content and AsA/DHA ratio and decreased DHA content in LT stressed mung bean seedlings, compared to the seedlings exposed to LT only (Figure 4A–C). Exogenous Spd also increased GSH/GSSG ratio by increasing GSH content and reducing GSSG content in LT affected seedlings (Figure 4D–F).

#### 2.6.2. Ascorbate-Glutathione (AsA-GSH) Cycle Enzymes

**Figure 5:**
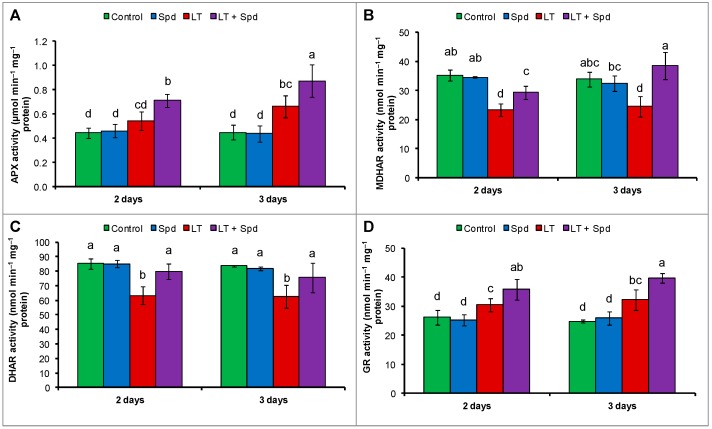
Activities of ascorbate peroxidase (APX) (**A**); monodehydroascorbate reductase (MDHAR) (**B**); dehydroascorbate reductase (DHAR) (**C**) and glutathione reductase (GR) (**D**) in mung bean seedlings. Spd and LT indicate spermidine (0.25 mM) and low temperature (6 °C), respectively. Mean (±SD) was calculated from three replicates for each treatment. Bars with different letters are significantly different at *p* ≤ 0.05 applying DMRT.

Compared to the control, APX activity increased after three days of LT stress. No significant change was observed in APX activity after two days, compared the control (Figure 5A). Activities of MDHAR and DHAR reduced both after two days and three days of LT stress (Figure 5B,C) (compared to the control). GR activity increased slightly after two days and three days of LT stress, compared to the control (Figure 5D). Exogenous Spd was very effective at restoring and increasing the activities of all the enzymes of the AsA-GSH cycle, including APX, MDHAR, DHAR and GR (Figure 5A–D).

### 2.7. Activities of Catalase (CAT) and Glutathione Peroxidase (GPX)

**Figure 6:**
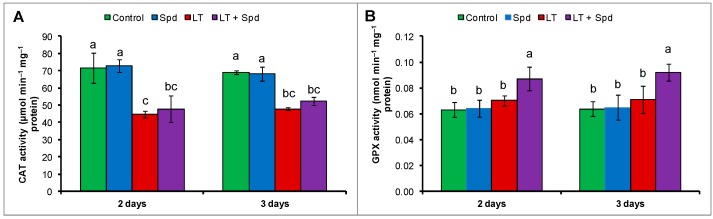
Activities of catalase (CAT) (**A**) and glutathione peroxidase (GPX) (**B**) in mung bean seedlings. Spd and LT indicate spermidine (0.25 mM) and low temperature (6 °C), respectively. Mean (±SE) was calculated from three replicates for each treatment. Bars with different letters are significantly different at *p* ≤ 0.05 applying DMRT.

Catalase activity reduced under LT stress and GPX activity was not affected by LT, compared to the control. Exogenous Spd application did not increase or affect the activity of CAT but increased the activity of GPX, compared to the LT treatment only (Figure 6A,B).

### 2.8. Glyoxalase System and Methylglyoxal (MG) Detoxification

**Figure 7:**
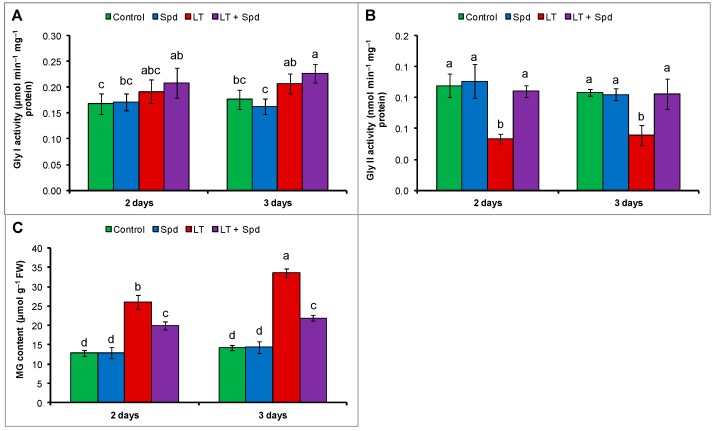
Activity of glyoxalase I (Gly I) (**A**) and glyoxalase II (Gly II) (**B**); and methylglyoxal (MG) content (**C**) in mung bean seedlings. Spd and LT indicate spermidine (0.25 mM) and low temperature (6 °C), respectively. Mean (±SD) was calculated from three replicates for each treatment. Bars with different letters are significantly different at *p* ≤ 0.05 applying DMRT.

Activity of Gly I did not change under LT stress (compared to the control) and exogenous Spd failed to increase its activity significantly in LT affected seedlings (compare to LT affected seedling without Spd application) (Figure 7A). Gly II activity decreased under LT stress of both durations compared the control. When the LT affected mung bean seedlings were pretreated with exogenous Spd those increased Gly II activity, compared to the seedlings without Spd under LT stress (Figure 7B). The MG toxicity under LT stress is evident from increased MG content by 103% after two days and 136% after three days of LT stress, respectively, compared to the control seedlings. MG content of Spd pretreated mung bean seedlings decreased by 23% and 35% after two days and three days of LT stress, respectively, compared to the LT stress without Spd (Figure 7C).

### 2.9. Endogenous Polyamines (PAs) Pool

**Figure 8:**
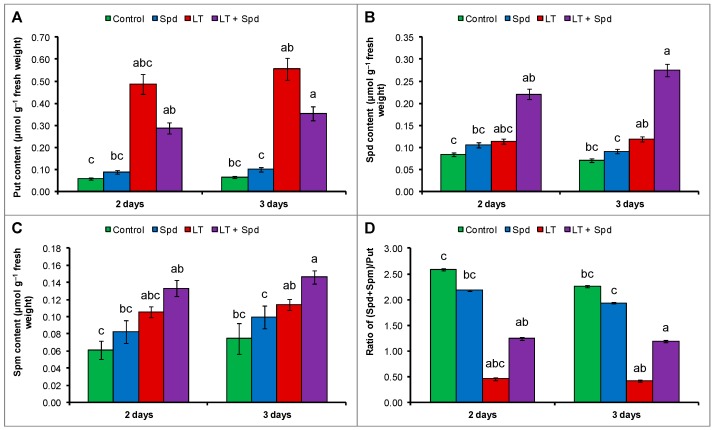
Endogenous putrescine (Put) content (**A**); spermidine (Spd) content (**B**); spermine (Spm) content (**C**) and the ratio of (Spd + Spm)/Put (**D**) in mung bean seedlings. Spd and LT indicate exogenously applied spermidine (0.25 mM) and low temperature (6 °C), respectively. Mean (±SD) was calculated from three replicates for each treatment. Bars with different letters are significantly different at *p* ≤ 0.05 applying DMRT.

The endogenous Put level increased highly under LT stress whereas, the endogenous Spd and Spm increased slightly under LT stress which decreased the (Spd + Spm)/Put, compared to the control treatment (Figure 8A–D). Exogenous Spd supplementation in LT affected seedlings did not change the Put content significantly but increased the endogenous Spd and Spm levels, which increased the ratio of (Spd + Spm)/Put, compared to the LT treatment only (Figure 8A–D).

## 3. Discussion

Low temperature injury in mung bean seedlings is reflected by growth reduction, which is evidenced by decreased seedlings dry weight (DW) (Figure 1A). Lin and Saltveit [17] reported the growth reduction in mung bean plants under LT stress. Low temperature stress inhibits various metabolic reactions, which are often expressed by different phenotypic symptoms including growth reductions [18]. Soil and plant water relationship, photosynthesis, and translocation of assimilates are impaired under LT stress, which reduce growth [19]. Thus, plants show different morphological injury symptoms like stunted plant, bushy plants, early maturity, and yellowing of leaves under LT stress [20]. When mung bean seedlings were pretreated with Spd and after that subjected to LT they exhibited reduced injury in terms of growth and the seedlings had higher DW, compared to the non-pretreated seedlings under LT (Figure 1A). PAs improve chl content; PSII phytochemistry efficiency; protect cell organelles from oxidative damage; and regulate metabolites, hormones and signaling molecules related to plant development and stress response [8,14,21].

Dehydration stress is also associated with LT stress that amplifies ROS generation. Dehydration stress is often associated with higher level of compatible solutes within plant cells because water stress induces plants to accumulate osmoprotectants or compatible solutes [22,23,24]. Mung bean plants exposed to LT showed a reduction in leaf RWC with higher accumulation of leaf Pro level (compared to that of control treatment), which is an indication of LT-induced dehydration stress (Figure 1B,C). A similar decrease of leaf RWC and Pro accumulation was reported in previous studies with LT stress [22,23]. Inhibition of water uptake through roots and shoots due to declined metabolic activity and interruption of leaf transpiration is a common phenomenon causing water or dehydration stress in LT affected plants [23]. Free proline acts as an osmoprotectant, antioxidant and maintains the cellular environment suitable for different metabolic processes, which enhances stress adaptation [2,25]. Proline accumulation was reported to be correlated to stress tolerance. Alet *et al.* [22] reported that Put accumulation in *Arabidopsis thaliana* transgenic lines was associated with higher Pro accumulation and enhanced tolerance to dehydration and freezing stress. In our study, overproduction of Pro was noticed after Spd supplementation in LT affected mung bean plants (Figure 1B), which corroborates the higher leaf RWC (Figure 1C) and indicates relaxation of dehydration stress.

Reduction of chl biosynthesis and rapid degradation of chl are common occurrence and reason for diminished chl content under environmental stresses including LT stress [23,26]. Reduction of leaf chl due to LT (Figure 1D) was restored by exogenous application in Spd. PAs can enter the chloroplast to scavenge excess ROS. PAs prevent stress-induced loss of protein and enzymes, and protects chloroplasts and photosynthetic pigments [14,27]. Exogenous Spd application inhibited the degradation of photosynthetic pigments including chl and carotenoid in ginger plants under LT stress [14]. Reduction of photosynthetic pigment content, inhibition of photochemistry efficiency or unbalanced function of photosystem II (PSII), inhibition of enzymatic activities under LT stress might be associated with ROS generation. ROS are responsible for damaging biomolecules causing peroxidation of lipid and oxidation of fatty acid. ROS injuring biomembranes including cellular, chloroplast and thylakoid membrane disrupts cellular homeostasis [21]. In the present study, histochemical staining localized H_2_O_2_ and O_2_^•−^ at the tissue level in the leaves of the mung bean plants (Figure 2A,B). Stress induced ROS generation and its damage at the tissue level is common and was previously reported in other plants [28]. Low temperature induced ROS generation and membrane damage are also reflected from the increased H_2_O_2_ content and membrane lipid peroxidation (indicated by higher MDA content). Exogenous Spd application reduced oxidative damage in mung bean seedlings after both two and three days (Figure 2 and Figure 3). Li *et al.* [14] reported that exogenous Spd increased ginger seedlings tolerance to LT stress protecting PSII from damage, keeping high level of unsaturated fatty acid and enhancing ROS detoxification process. Reduction of ROS induced oxidative stress by exogenous PAs application was reported in other studies under different abiotic stresses [10,29].

Ascorbate is a vital water-soluble antioxidant of plant cell reacting with a range of ROS including H_2_O_2_, O_2_^•−^ and ^1^O_2_. AsA also scavenges OH^•^ at diffusion-controlled rates [30]. Besides acting as scavenger of ROS, AsA plays roles in maintaining reduced state of chloroplastic antioxidant, α-tocopherol. AsA takes part in synthesizing zeaxanthin that dissipates excess light energy in the thylakoid membranes and thereby prevent oxidative damage [31]. AsA also keeps prosthetic metal ions in a reduced form and thus increase efficiency of antioxidant enzyme activities [32]. In the present study, AsA content reduced under LT that was responsible for increasing ROS generation and oxidative stress. However, exogenous Spd addition increased AsA content in LT affected plants and reduced the oxidative stress (Figure 2 and Figure 3).

Scavenging different ROS, GSH acts as a potential antioxidant [33]. GSH helps in regenerating other potential water-soluble antioxidants like AsA through the AsA-GSH cycle [34]. GSH sustains the reduced state of α-tocopherol and zeaxanthin. GSH, by preventing the oxidation of protein thiol groups, prevents denaturation of proteins. Acting as the substrate for GPX, GSH remove ROS [35]. It acts as a storage compound and transport of reduced sulfur in the plant cell [36]. The ratio of GSH/GSSG functions in redox signaling pathways [37]. Interacting with hormones, redox molecules participates in signal transduction [38,39]. GSH serves important functions and contribute to regulation of plant development, cell cycle, gene expression, and protein activity both under normal and stress conditions [40,41]. Low temperature stress slightly increased the GSH content in mung seedlings and reduced the GSH/GSSG ratio, which partly contributed oxidative stress. Exogenous Spd, when applied to LT affected seedlings, increased the GSH content and GSH/GSSG ratio, which helped to reduce oxidative stress and is reflected in reduced H_2_O_2_ and MDA levels (Figure 3A,B and Figure 4D,F). These results are supported by the results of the previous study. Exogenous Spd significantly increased AsA and GSH contents as well as AsA/DHA and GSH/GSSG ratios in ginger plants under LT stress, which reduced oxidative damage and boosted tolerance to LT stress [14].

APX scavenges H_2_O_2_ using AsA as substrate. In the AsA-GSH cycle, APX uses two molecules of AsA to reduce H_2_O_2_ to water, and, during this reaction, MDHA is generated. MDHA is a radical with a short life span and can disproportionate into DHA and AsA, which is catalyzed by MDHAR or ferredoxin in a water–water cycle in the chloroplast, where NADPH (reduced nicotinamide adenine dinucleotide phosphate) acts as electron donor [42]. DHA can also be reduced to AsA by DHAR, which uses GSH as the reductant [43]. Reduction of AsA content with the increase of DHA content in the LT affected mung bean indicates cellular oxidative stress. The decrease of AsA content was responsible for the decrease in AsA/DHA ratio (Figure 4A,C). The reduction of AsA content was due to higher APX activity and reduced MDHAR and DHAR activities, which are involved in recycling of AsA (Figure 5A–C) [44]. However, exogenous Spd application increased the MDHAR and DHAR activities and increased the contents of AsA in LT affected mung bean seedlings. Increase of AsA content was also associated with increased AsA/DHA ratio (Figure 4A,C). The increased AsA content and AsA/DHA ratio reduced oxidative stress (Figure 3A,B). During scavenging ROS, GSH is oxidized to GSSG. The oxidized GSSG is recycled by the activity of GR in the AsA-GSH cycle. The results of the present study show that the GSH and GSSG content and GR activity increased, but GSH/GSSG ratio decreased in LT affected mung bean seedlings, compared to the control. Moreover, Spd pretreatment increased GSH content and GR activity in LT affected seedlings (Figure 5D). GR catalyzes the NADPH-dependent reduction of disulfide bond of GSSG and regenerates GSH; thus it is important for maintaining the GSH pool [45]. So, increase of GR activity maintained the GSH content high and helped to increase the ratio of GSH/GSSG, which is vital for accelerating the H_2_O_2_ scavenging pathway [46]. Li *et al.* [14] reported that exogenous Spd stimulated the activities of antioxidant enzymes of AsA-GSH cycle in ginger plant under LT stress, which include APX, DHAR, MDHAR and GR activities. Enhancement of antioxidant enzymes imparted LT tolerance to those seedlings [14]. Similar results have been demonstrated in experiments on the treatment of plants using PAs under salt stress [10,21,29].

CATs are heme-containing enzymes involved in direct dismutation of H_2_O_2_ into H_2_O and O_2_ [47]. CAT activity of LT stressed mung bean seedlings decreased and exogenous Spd pretreatment did not increase its activity further in LT-affected seedlings (Figure 6A). Higher GPX activity reduces cellular oxidative stress in different ways. GPX uses GSH to reduce H_2_O_2_, organic and lipid hydroperoxides (LOOHs) [35]. GPX is considered as principal cellular enzyme capable of preventing oxidative stress, membrane damage and enhancing repairing of membrane [48]. Low temperature increased the GPX activity significantly, compared to the control (Figure 6B). Exogenous Spd application in LT affected seedlings increased GPX activity, compared to the LT-affected seedlings without Spd (Figure 6B). Increased GPX activity was involved in reducing H_2_O_2_, and organic and LOOHs [35] that decreased the oxidative stress of mung bean seedlings (Figure 3A,B). The activity of GPX in the presence of Spd under LT stress was also higher in ginger plant than those under LT stress alone as reported by Li *et al.* [14].

Increase of MG toxicity in LT affected mung bean seedlings was reversed by exogenous Spd. The reduction in MG toxicity is due to the enhanced activities of the glyoxalase enzymes and increased GSH level after Spd pretreatment (Figure 4D and Figure 7A–C) as the enzymes Gly I and Gly II detoxify MG in step-by-step reactions using GSH [49]. Polyamine-induced regulation of MG levels was studied in animal systems but was rarely studied in plant system [50].

Accumulation and modulation of PAs are common plant stress response and are considered as vital for stress adaptation [10,21,29]. Mung bean seedlings exposed to LT stress accumulated higher amount of free PAs, including Put, Spd and Spm, compared to the control (Figure 8A–D). A higher accumulation of Put with decrease in (Spd + Spm)/Put ratio (Figure 8D) indicates cytotoxicity under LT stress [14]. LT-affected seedlings supplemented with exogenous Spd modulated Put, Spd and Spm that contribute to a higher (Spd + Spm)/Put ratio (Figure 8A–D). This is considered a reduction in cellular toxicity [14]. The roles of exogenous Spd in regulating endogenous PAs was studied and reported under different stresses, which indicate possible interlink/signaling function of PAs [14].

## 4. Experimental Section

### 4.1. Plant Materials and Growth Condition

Seeds of mung bean (*Vigna radiata* L. cv. BARI Mung-3) plants were placed in a petri dish containing six layer of moist filter paper and kept in germinator in dark place for three days. Then, germinated seedlings were transferred into growth chamber under controlled conditions (light, 350 μmol·photon·m^−2^·s^−1^; temperature, 25 ± 2 °C; relative humidity, 65%–70%); 10,000-fold diluted Hyponex solution (Hyponex, Osaka, Japan) was applied as nutrient. Two sets of four-day-old seedlings were pretreated with Spd (0.25 mM) for 24 h. These pre-treated seedlings were then exposed to 6 °C temperature at the fifth day. Other two sets of seedlings were exposed to 6 °C temperature (without Spd pretreatment). Control seedlings were grown with Hyponex solution. Another two sets of seedlings were grown with Spd without any stress. All treatments were considered for 2 and 3 days and after that data on different parameters were taken following the standard methodology.

### 4.2. Measurement of Growth Parameter

Plant height was taken from each set of seedlings and expressed as cm. Ten randomly selected fresh seedlings from each treatment were dried at 80 °C for 48 h, then weighed and considered as dry weight (DW), which was expressed in gram (g).

### 4.3. Measurement of Relative Water Content

Relative water content (RWC) of leaf was measured according to Barrs and Weatherly [51]. Fresh weight (FW), turgid weight (TW) and dry weight (DW) of leaves were taken, RWC was calculated by following formula:

$$\mathrm{RWC}\;(\%)=\frac{\mathrm{FW}-\mathrm{DW}}{\mathrm{TW}-\mathrm{DW}}\times 100$$

### 4.4. Measurement of Chlorophyll Content

Leaves supernatant were extracted with 80% *v*/*v* acetone (centrifuging at 5000× *g*), absorbances were taken with UV-visible spectrophotometer at 663 and 645 nm for chl *a* and chl *b* content, respectively; chl contents were calculated according to Arnon [52].

### 4.5. Measurement of Proline Content

Proline (Pro) was appraised according to Bates *et al.* [53]. Leaves were homogenized in 3% sulfosalicylic acid, centrifuged at 11,500× *g*. Filtrate was mixed with acid ninhydrin with glacial acetic acid and phosphoric acid. After incubating the mixture at 100 °C for 1 h and cooling, toluene was added, after several minutes chromophore containing toluene was read spectrophotometrically at 520 nm.

### 4.6. Histochemical Detection of Hydrogen Peroxide and Superoxide

The H_2_O_2_ and O_2_^•−^ were localized histochemically [28] by staining leaves with 1% 3,3-diaminobenzidine (DAB) and 0.1% nitroblue tetrazolium (NBT) solution, respectively. Leaves were immersed in those solutions until brown spots appeared due to the reaction of DAB with H_2_O_2_ or dark blue spots appeared due to the reaction of NBT with O_2_^•−^. After that, leaves were blanched in boiling ethanol to observe the spots.

### 4.7. Measurement of Lipid Peroxidation

The level of lipid peroxidation was measured by estimating MDA, using thiobarbituric acid (TBA) according to Heath and Packer [54] with modifications [49].

### 4.8. Measurement of Hydrogen Peroxide Content

Hydrogen peroxide was assayed according to Yu *et al.* [55] extracting leaves in potassium-phosphate buffer (pH 6.5) (centrifuging at 11,500× *g*), then reacting with a mixture of TiCl_4_ in 20% H_2_SO_4_ (*v*/*v*) and was measured spectrophotometrically at 410 nm.

### 4.9. Measurement of Methylglyoxal Level

Leaves were homogenized in 5% perchloric acid and centrifuged at 4 °C for 10 min at 11,000× *g*. The supernatant was decolorized by adding charcoal. The supernatant was neutralized by saturated solution of potassium carbonate at room temperature. The neutralized supernatant was used for MG estimation by adding sodium dihydrogen phosphate and *N*-acetyl-l-cysteine to a final volume of 1 mL. Formation of the product *N*-α-acetyl-*S*-(1-hydroxy-2-oxo-prop-1-yl)cysteine was recorded after 10 min at a wavelength of 288 nm according to Wild *et al.* [56]. The MG content within the plant sample was calculated by using standard curve of known concentration of MG and expressed as µmol·g^−1^ FW.

### 4.10. Extraction and Measurement of Ascorbate and Glutathione

Leaves (0.5 g) were homogenized in 5% meta-phosphoric acid containing 1 mM EDTA (centrifuged at 11,500× *g*) for 15 min at 4 °C and the supernatant was collected for analysis of ascorbate and glutathione. Ascorbate content was determined following the method of Huang *et al.* [57] with some modifications as described by Hasanuzzaman *et al.* [49]. The glutathione pool was assayed according to previously described methods [55] with modifications as described by Paradiso *et al.* [58] and Hasanuzzaman *et al*. [49]. Standard curves with known concentrations of GSH and GSSG were used. The content of GSH was calculated by subtracting GSSG from total GSH.

### 4.11. Protein Determination

The protein concentration of each sample was determined following the method of Bradford [59] using BSA (bovine serum blood) as a protein standard.

### 4.12. Enzyme Extraction and Assays

Leaves were homogenized in K-P buffer (pH 7.0) containing KCl, ascorbate, β-mercaptoethanol and glycerol. Homogenates were centrifuged at 11,500× *g*, supernatants were assayed.

CAT (enzyme commission number, EC: 1.11.1.6) activity [49]: Decrease of absorbance (by decomposition of H_2_O_2_) at 240 nm was recorded for 1 min. Reaction was initiated with enzyme extract; activity was calculated from extinction coefficient 39.4 M^−1^·cm^−1^.

APX (EC: 1.11.1.11) activity [60] assay: Reaction buffer solution contained 50 mM K-P buffer (pH 7.0), 0.5 mM AsA, 0.1 mM H_2_O_2_, 0.1 mM EDTA, and enzyme extract (final volume 700 μL). Reaction was started by adding H_2_O_2_. Activity was measured at 290 nm for 1 min using an extinction coefficient 2.8 mM^−1^·cm^−1^.

MDHAR (EC: 1.6.5.4) activity [61]: Reaction mixture contained 50 mM Tris-HCl buffer (pH 7.5), 0.2 mM NADPH, 2.5 mM AsA, 0.5 unit of AO and enzyme solution (final volume 700 μL). Reaction was started by adding AO. Absorbance was taken at 340 nm; activity was calculated from change in for 1 min using an extinction coefficient of 6.2 mM^−1^·cm^−1^.

DHAR (EC: 1.8.5.1) activity [60]: Reaction buffer contained 50 mM K-P buffer (pH 7.0), 2.5 mM GSH, and 0.1 mM DHA. Activity was calculated from change in absorbance at 265 nm for 1 min using extinction coefficient of 14 mM^−1^·cm^−1^.

GR (EC: 1.6.4.2) activity [49]: Reaction mixture contained 0.1 M K-P buffer (pH 7.0), 1 mM EDTA, 1 mM GSSG, 0.2 mM NADPH; enzyme solution (final volume 1 mL). Reaction was initiated with GSSG; decrease in absorbance at 340 nm was recorded for 1 min (calculated using extinction coefficient 6.2 mM^−1^·cm^−1^).

GPX (EC: 1.11.1.9) activity was measured as described by Elia *et al.* [62] with slight modification as described by Hasanuzzaman *et al.* [49]. The reaction mixture consisted of 100 mM K-P buffer (pH 7.0), 1 mM EDTA, 1 mM NaN_3_, 0.12 mM NADPH, 2 mM GSH, 1 unit GR, 0.6 mM H_2_O_2_ (as a substrate) and 20 μL of sample solution. The oxidation of NADPH was recorded at 340 nm for 1 min and the activity was calculated using the extinction coefficient of 6.62 mM^−1^·cm^−1^.

Glyoxalase I (EC: 4.4.1.5) [49]: Assay mixture contained 100 mM K-P buffer (pH 7.0), 15 mM magnesium sulphate, 1.7 mM GSH and 3.5 mM MG (final volume 700 μL). Reaction was started by adding MG; increase in absorbance was recorded at 240 nm for 1 min. Activity was calculated using extinction coefficient 3.37 mM^−1^·cm^−1^.

Glyoxalase II (EC: 3.1.2.6) [63]: Formation of GSH at 412 nm was monitored in 1 min. Reaction mixture contained 100 mM Tris–HCl buffer (pH 7.2), 0.2 mM DTNB, 1 mM *S*-d-lactoylglutathione (SLG) (final volume of 1 mL). Reaction was started by SLG; activity was calculated using extinction coefficient 13.6 mM^−1^·cm^−1^.

### 4.13. Measurement of Free Polyamine Content

Endogenous free PAs were estimated according to Kotzabasis [64]. In brief, leaf tissue (0.1 g) was homogenized in 1 mL of 5% (*v*/*v*) cold perchloric acid (PCA). The homogenates were kept at 2 °C for 2 h and centrifuged at 15,000× *g* for 20 min. The supernatant was collected and stored at 2 °C. Aliquots (200 μL) of supernatant was mixed 1:1 (*v*/*v*) with 12 N HCL and hydrolyzed for 16 h at 110 °C in flame sealed ampoules. The hydrolyzed products were centrifuged at 3000× *g* to remove carbonized material and then evaporated at 70 °C. The dried pellet was redissolved in 200 μL of 5% perchloric acid. The nonhydrolyzed PCA supernatant containing free PAs was subjected to benzoylation in alkaline medium. The benzoyl-PAs were extracted with diethyl ether and then evaporated to dryness in a water bath. The benzoyl-PAs were redissolved in methanol and free PAs were analyzed by HPLC with a UV-vis spectrophotometric detector at 254 nm, and then determined using a standard curve of PAs (Put, Spd and Spm) and expressed as µmol·g^−1^·FW.

### 4.14. Statistical Analysis

All data obtained were subjected to analysis of variance (ANOVA) and the mean differences were compared by a Duncan’s multiple range test (DMRT) using XLSTAT v.2010 software [65]. Differences at *p* < 0.05 were considered significant.

## 5. Conclusions

The present study investigates the roles of exogenous Spd in enhancing AsA-GSH cycle components in improving the LT stress tolerance of mung bean seedlings. Mung bean seedlings exposed to LT were severely injured by oxidative stress, those seedlings showed chl break down, dehydration stress and these injury symptoms were also reflected through reduced seedlings growth or seedlings DW. Exogenous Spd improved the biochemical and physiological status of the LT affected mung bean seedlings. Maintenance of water status and alleviation of dehydration stress was conferred by exogenous Spd through osmotic regulation. Spd protected chl from oxidative damage or breakdown. Reduction of oxidative damage in mung bean seedlings was the prime advantageous effect among the studied parameters of this experiment, which was due to the enhanced AsA-GSH cycle components including AsA and GSH contents and activities of APX, MDHAR, DHAR and GR. The activity of GPX also increased by Spd pretreatment of LT affected seedlings. Previous studies revealed the diversified roles of PAs, which improve plant performance in both non-stress and stress environments. PAs may form complexes with antioxidant enzymes that function more efficiently than the isolated enzyme [9]. PAs could induce synthesis antioxidant metabolites like AsA, GSH, polyphenols and flavonoids having differential metabolic function [10,21,66]. PAs may act as central signaling molecules, which could boost AsA-GSH recycling in enhancing plants tolerance to abiotic stresses [9,10,21,67]. Induction of AsA and GSH or AsA/DHA and GSH/GSSG by exogenous Spd indicates Spd function in the AsA-GSH cycle as ROS scavenger in the present study. PAs showed advantageous effects in other physiological processes in different studies, such as transmitting ROS and hormones signals, cell division, cell differentiation and activating enzymes and expression of stress responsive gene [3]. Similar advantageous roles of exogenous Spd might allow mung bean seedlings to survive and perform better under LT. Spermidine induced reduction of MG toxicity by enhancing glyoxalase system opens a new door towards research regarding PAs, as few studies with glyoxalase pathway and PAs have been reported.

## Supplementary Materials

Supplementary materials can be found at http://www.mdpi.com/1422-0067/16/12/26220/s1.
