# Supplementary material for: Exogenous Spermidine Alleviates Low Temperature Injury in Mung Bean (Vigna radiata L.) Seedlings by Modulating Ascorbate-Glutathione and Glyoxalase Pathway
Source: Int J Mol Sci. 2015 Dec 17;16(12):30117–32. doi: 10.3390/ijms161226220 (PMC4691163; doi:10.3390/ijms161226220)
Supplement: Supplementary file 1 [file ijms-16-26220-s001.pdf]

Supplementary Materials: Exogenous Spermidine Alleviates Low Temperature Injury in Mung Bean (*Vigna radiata* L.) Seedlings by Modulating Ascorbate-Glutathione and Glyoxalase Pathway

Kamrun Nahar, Mirza Hasanuzzaman, Md. Mahabub Alam and Masayuki Fujita

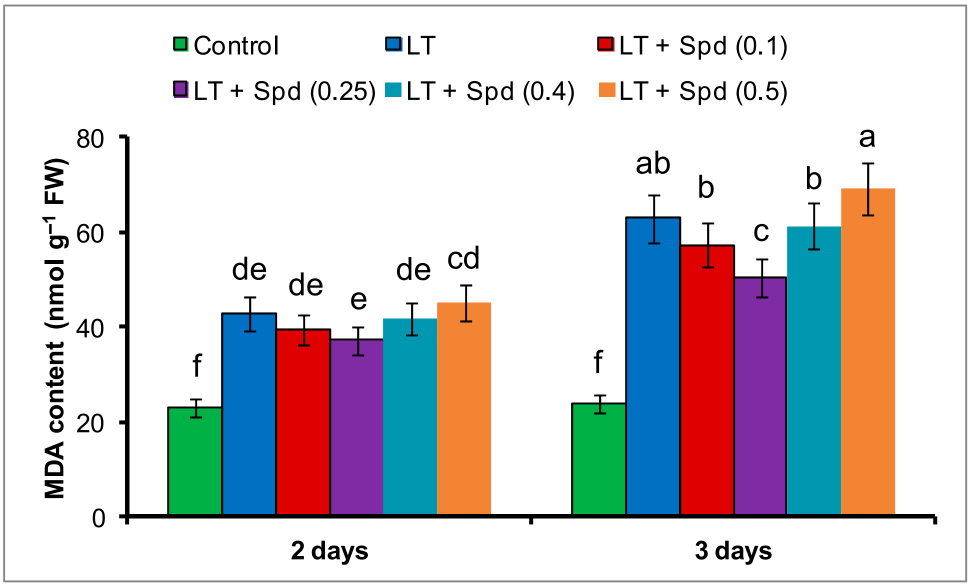

Figure S1. MDA (malondialdehyde, a product of lipid peroxidation) content in mung bean seedlings. Spd and LT indicate spermidine and low temperature (6 °C) respectively. Here different concentration of Spd such as, 0.1, 0.25, 0.4 and 0.5 mM was applied as pre-treatment on LT affected seedlings. Mean (±SE) was calculated from three replicates for each treatment. Bars with different letters are significantly different at $p \leq 0.05$ applying DMRT (Duncun's Multipple Range Test).
